# Diminished Endothelial Function but Normal Vascular Structure in Adults with Tetralogy of Fallot

**DOI:** 10.3390/jcm11030493

**Published:** 2022-01-19

**Authors:** Daniel Goeder, Renate Oberhoffer-Fritz, Leon Brudy, Laura Willinger, Michael Meyer, Peter Ewert, Jan Müller

**Affiliations:** 1 German Heart Centre Munich, Department of Paediatric Cardiology and Congenital Heart Disease, Technical University Munich, 80636 Munich, Germany; daniel.goeder@tum.de (D.G.); renate.oberhoffer@tum.de (R.O.-F.); leon.brudy@tum.de (L.B.); laura.willinger@tum.de (L.W.); michael.meyer@tum.de (M.M.); ewert@dhm.mhn.de (P.E.); 2 Institute of Preventive Pediatrics, Technical University Munich, 80992 Munich, Germany

**Keywords:** EndoPAT, endothelium, carotid intima-media thickness

## Abstract

The life expectancy of patients with Tetralogy of Fallot (ToF) has increased in recent years. As a result, other risk factors with later onset in life are in the focus of patient care. Endothelial function is an early indicator of cardiovascular risk and was investigated along further structural vessel properties. A total of 17 patients (41.7 ± 7.1 years, 8 women) with Tetralogy of Fallot were 1:2 matched for sex with 34 (38.9 ± 8.1 years, 16 women) healthy volunteers. Participants received an assessment of their endothelial function and a structural assessment of the aorta. Patients with ToF showed a reduced endothelial function determined by reactive hyperaemia index after adjusting for age, weight and height (ToF: 1.55 ± 0.31 vs. controls: 1.84 ± 0.47; *p* = 0.023). No differences in carotid intima-media thickness (cIMT) between the ToF and healthy controls (ToF: 0.542 ± 0.063 mm vs. controls: 0.521 ± 0.164 mm; *p* = 0.319) were found. Patients with ToF had reduced vascular function compared to healthy subjects. As the structural component is not affected, endothelial dysfunction seems not to have yet manifested itself as a morphological change. Nevertheless, long-term management of these patients should include vascular parameters.

## 1. Introduction

Most patients with congenital heart disease reach adulthood, resulting in a new challenge for cardiologists in terms of management, for caregivers in terms of resource utilization and for the patients themselves in terms of prevention of sequels [1,2,3]. In this context, the prevention of cardiovascular disease becomes imperative [4,5].

The arterial vessel and its ability to dilate and recoil in response to a variety of physical and chemical stimuli plays an important role in the onset and progression of cardiovascular disease. The endothelium, a single layer of squamous cells lining the inner surface of the arterial vessels, is the key regulator of the vascular tone. Its function can be measured noninvasively by brachial flow-mediated dilation or peripheral artery tonometry.

In adults with congenital heart disease (ACHD), some diagnostic groups are well studied in terms of their cardiovascular squeals. Patients with Fontan circulation [6,7,8,9] are known to have increased systemic vascular resistance, hypoxia and increased sympathetic activity that can result in altered endothelial function. A total of 50% of patients aged between 30 years and 40 years with coarctation of the aorta [10,11] are hypertensive [12], and endothelial dysfunction is a possible precursor. On the other hand, cyanotic CHD patients [13,14] have low total cholesterol and low-density lipoprotein levels. However, they also present endothelial dysfunction due to diminished numbers of endothelial progenitor cells and limited response to endothelin. However, research findings are still sparse, and the evidence supporting them is low.

The aim of this study was to investigate whether adults with Tetralogy of Fallot (TOF) have an impaired endothelial function, as measured by the reactive hyperemia index (RHI), or an abnormal cardiovascular structure, measured by the intima-media thickness of the common carotid artery (cIMT), when compared to healthy controls.

## 2. Materials and Methods

From April 2018 to October 2019, 17 patients (41.7 ± 7.1 years, 8 women) with Tetralogy of Fallot matched with 34 (38.9 ± 8.1 years, 16 women) healthy volunteers were prospectively enrolled into the study. After written informed consent was obtained, patients received an assessment of their endothelial function and a structural assessment of the aorta. All participants (ToF and controls) were free from syndromes and acute illnesses. NYHA class was I or II, and none took hypertensive agents. Patient characteristics are displayed in Table 1.

The study was approved by the local ethics board of the Technical University of Munich (project number: 108/18 S) and is part of the CARING (Cardiovascular Risk in Grown-up Congenital Heart Disease) project, which is registered in the “German Clinical Trial Registry” (DRKS-ID: DRKS00015248).

### 2.1. Endothelial Function

Endothelial function was assessed using the EndoPAT device (Itamar Medical, Caesarea, Israel). The subjects were examined in the morning, sober, in a darkened room. They were instructed not to drink any caffeine or alcohol and not to smoke for 12 h before the examination. Upon arrival at the clinic, each subject was given a 15 min rest period with subsequent blood pressure measurement in a supine position. The endothelial function was indirectly derived from the pulsative flow of the index finger. First, the pulsative flow was measured basally. Then, the blood pressure cuff placed on the right upper arm was inflated to a pressure of 200 mm/Hg, creating an occlusion for 5 min. In the subsequent post-occlusion phase, the diameter was observed once again for 2 min. The basis of this examination was the post-occlusive hyperemia-induced nitric-oxide-mediated vasodilatation. The primary outcome was described as a reactive hyperemia index (RHI) of the change from basal to post-occlusive phase. The inter-day reproducibility and intrasubject variability of RHI was moderate but slightly less with flow-mediated dilation using brachial artery ultrasound scanning. However, the EndoPAT method has the advantage of being less operator dependent [15,16].

### 2.2. Intima-Media Thickness

**Figure 1:**
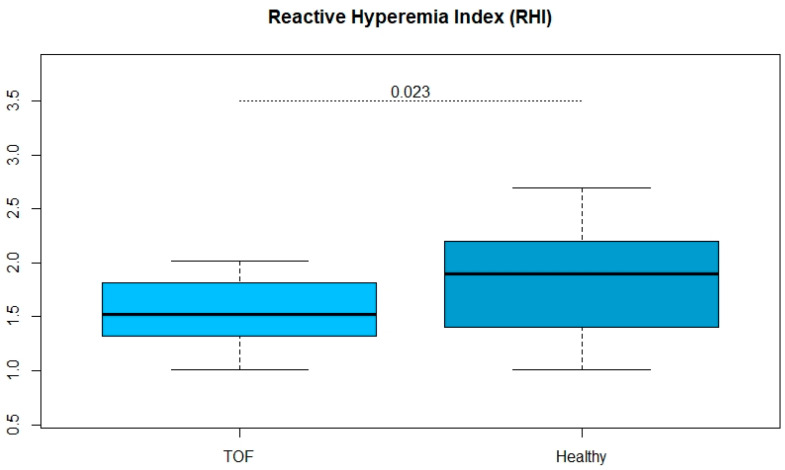
Box and whisker plot displaying multiple linear regression model adjusted for age, weight and height comparing reactive hyperemia index in healthy volunteers and patients with Tetralogy of Fallot.

Vascular structure was determined by measuring intima-media thickness of the common carotid artery (cIMT) as previously described [17,18]. In brief, all measurements were performed using the Cardiohealth Station of Panasonic (Yokohama, Japan), a semiautomated ultrasound system. With B-Mode, 4 measurements of cIMT at the far wall of the common carotid artery, 1 cm distal to the bulb, were recorded, and a mean value was calculated and used for all following analyses (Figure 1). The central frequency of the linear-array transducer was 8.9 MHz. Short-term and 1-week inter- and intra-operator cIMT measurement variability of the Cardiohealth Station is acceptable in healthy, young to middle-aged adults [19].

### 2.3. Data Analysis

Descriptive data are reported as mean ± SD in case of continuous variables and as counts (%) in case of categorical variables. Comparisons of demographic and clinical variables of patients with ToF and healthy controls were performed by independent T-test after matching 1:2 for sex.

The primary outcome of the study was the comparison of RHI between ToF and healthy controls that was performed by a multiple linear regression model adjusting for slight differences in age, height and weight. The secondary outcome was cIMT analyzed with the same methods and covariates. All data were analyzed using IBM SPSS Statistics 25. The significance level was set at <0.05 for all statistical tests.

## 3. Results

**Figure 2:**
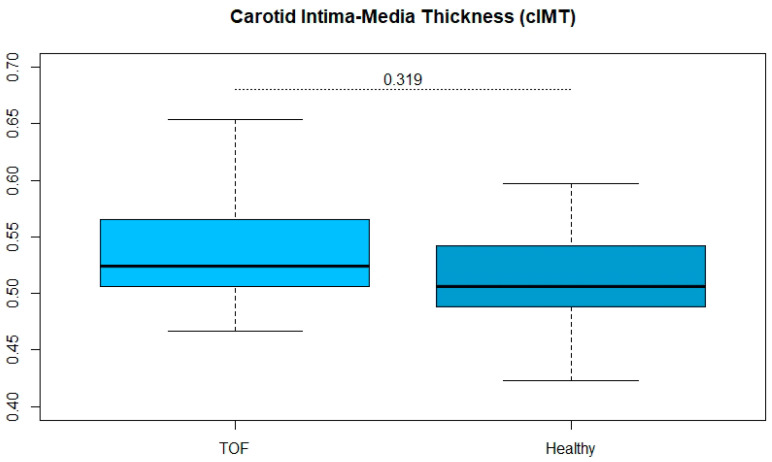
Box and whisker plot displaying multiple linear regression model adjusted for age, weight and height, comparing carotid intima-media thickness in mm in healthy volunteers and patients with Tetralogy of Fallot.

Patients with ToF showed a reduced endothelial function determined by reactive hyperaemia index after adjusting for age, weight and height (ToF: 1.55 ± 0.31 vs. controls: 1.84 ± 0.47; *p* = 0.023). There were no significant differences in cIMT between the ToF and healthy controls (ToF: 0.542 ± 0.063 mm vs. controls: 0.521 ± 0.164 mm; *p* = 0.319). Results are illustrated in Figure 1 and Figure 2.

## 4. Discussion

In this study, we found a reduced RHI, which suggests a reduced vascular function in the patient group compared to the healthy peers. In contrast, no significant changes were found in the structure of the carotid vessels. One possible explanation is that, in patients with ToF, there is a disturbance of vascular regulation, which has not yet manifested itself as a morphological change.

Arterial vessels have recently received more and more attention in patients with CHD in general, but specific diagnostic subgroup analyses have not been pursued so far. Those that do exist focus on more prominent heart defects, such as coarctation of the aorta [10,11,20,21], because it is already known that these patients develop high blood pressure. Alternatively, they focus on patients with Fontan circulation [6,8,9,22] because of low venous pressure and impaired fibrinolysis.

In patients with ToF, there is only a small study [23] of eleven children and a small control group that suggests that children with ToF, already at a young age, experience changes in vascular function and structure. In our study, we can only confirm these results on one point, as vascular function of the microcirculation was also impaired in our investigation. Although different methods were used in our study (flow-mediated dilation [23] and peripheral arterial tonometry), the results remained the same. It suggests that there is a generalized vascular disturbance of the microcirculation in patients with ToF, independent of age. Multifactorial reasons come into play here. Endothelial dysfunction might be related to postoperative hemodynamic abnormalities, such as changes in transmural pressure [24,25], flow patterns [22] and structure of smooth muscle cells of the aortic wall [23]. Lifestyle factors, such as impaired physical activity and obesity, are also always discussed in this context. Unfortunately, this study does not address physical activity behavior, but recent reports suggest that there is hardly any difference between patients with CHD and healthy peers in general [26,27].

Interestingly, the endothelial dysfunction seems not to translate into results in morphological change of the vessel structure, as cIMT is comparable to healthy controls. Naturally, this should be the case, as endothelial dysfunction should also affect the structure of the walls and translate into a thicker cIMT. However, it should be stated that functional limitation at the endothelium precedes a thickening of the wall structures. Since the participants in our study are still young, the wall structures may not yet be affected by means of cIMT. However, studies in children and in adults have not yet been able to show clearly that the wall structures (intima-media thickness) in a ToF collective were thicker [17,18]. Neither could a functional impairment of the vasculature be shown [28,29].

One possible reason for that could be that the sensitive measuring methods of microcirculation, which were designed for healthy people and often only validated on them, are simply not applicable to our patients with CHD. This would fortunately mean the micro- and macro-circulation and arterial structure would be “normal” in patients with ToF.

Nevertheless, much more attention should be paid to micro- and macro-circulation in patients with CHD in general [5] because endothelial dysfunction is an early marker for the development of atherosclerosis and occurs before the clinical manifestation of atherosclerosis. Since we are dealing with an aging cohort in this patient group, it will be of central interest in the future to detect subclinical parameters early and to counteract them with interventions.

### Limitations

The sample size of the ToF group is rather small. Although statistical models were used for their adjustments, there may still be possible bias. Future studies may be able to use the RHI values collected in this study, which were not reliably available, for a precise power calculation. It could therefore be that the lack of differences in IMT is simply due to an underpowered sample. Lifestyle factors and other risk factors play an important role in the development of endothelial dysfunction but were not structurally assessed in this report and were therefore omitted from the analysis.

## 5. Conclusions

Patients with ToF had reduced vascular function compared to healthy subjects. Since the IMT as the structural component was not affected, this disturbance of vascular regulation seems not to have yet manifested itself as a morphological change. Nevertheless, vascular parameters should be moved more into clinical focus to improve the long-term management of those patients in terms of their cardiovascular risk.

## Figures and Tables

**Table 1:** Subject characteristics.

Variable	Tetralogy of Fallot (MW ± SD)	Norm (MW ± SD)	*p*-Value
Sex (male/female)	9/8	18/16	-
Age (years)	41.7 ± 7.1	38.9 ± 8.1	0.432
Height (cm)	174.4 ± 4.6	171.8 ± 6.2	0.739
Weight (kg)	79.6 ± 14.5	73.9 ± 12.4	0.285

*t*-test for group comparison in age, height and weight.

## Data Availability

The data is available upon request from the corresponding author.

## References

[1] Tutarel O. (2014). Acquired heart conditions in adults with congenital heart disease: A growing problem. Heart.

[2] Baumgartner H., Budts W., Chessa M., Deanfield J., Eicken A., Holm J., Iserin L., Meijboom F., Stein J., Szatmari A. (2014). Recommendations for organization of care for adults with congenital heart disease and for training in the subspecialty of ‘Grown-up Congenital Heart Disease’ in Europe: A position paper of the Working Group on Grown-up Congenital Heart Disease of the European Society of Cardiology. Eur. Heart J..

[3] Triedman J.K., Newburger J.W. (2016). Trends in Congenital Heart Disease: The Next Decade. Circulation.

[4] Bauer U.M.M., Korten M.A., Diller G.P., Helm P., Baumgartner H., Ewert P., Tutarel O. (2019). Cardiovascular risk factors in adults with congenital heart defects—Recognised but not treated? An analysis of the German National Register for Congenital Heart Defects. Int. J. Cardiol..

[5] Muller J., Brudy L., Meyer M., Ewert P., Oberhoffer R. (2020). The cardiovascular burden of congenital heart disease—Not only in times of COVID-19. Int. J. Cardiol..

[6] Lambert E., d’Udekem Y., Cheung M., Sari C.I., Inman J., Ahimastos A., Eikelis N., Pathak A., King I., Grigg L. (2013). Sympathetic and vascular dysfunction in adult patients with Fontan circulation. Int. J. Cardiol..

[7] Binotto M.A., Maeda N.Y., Lopes A.A. (2008). Altered endothelial function following the Fontan procedure. Cardiol. Young.

[8] Jin S.M., Noh C.I., Bae E.J., Choi J.Y., Yun Y.S. (2007). Impaired vascular function in patients with Fontan circulation. Int. J. Cardiol..

[9] Mahle W.T., Todd K., Fyfe D.A. (2003). Endothelial function following the Fontan operation. Am. J. Cardiol..

[10] Radke R.M., Diller G.P., Duck M., Orwat S., Hartmann D., Thum T., Baumgartner H. (2014). Endothelial function in contemporary patients with repaired coarctation of aorta. Heart.

[11] de Divitiis M., Pilla C., Kattenhorn M., Zadinello M., Donald A., Leeson P., Wallace S., Redington A., Deanfield J.E. (2001). Vascular dysfunction after repair of coarctation of the aorta: Impact of early surgery. Circulation.

[12] Hager A., Kanz S., Kaemmerer H., Schreiber C., Hess J. (2007). Coarctation Long-term Assessment (COALA): Significance of arterial hypertension in a cohort of 404 patients up to 27 years after surgical repair of isolated coarctation of the aorta, even in the absence of restenosis and prosthetic material. J. Thorac. Cardiovasc. Surg..

[13] Cordina R.L., Nakhla S., O’Meagher S., Leaney J., Graham S., Celermajer D.S. (2015). Widespread endotheliopathy in adults with cyanotic congenital heart disease. Cardiol. Young.

[14] Oechslin E., Kiowski W., Schindler R., Bernheim A., Julius B., Brunner-La Rocca H.P. (2005). Systemic endothelial dysfunction in adults with cyanotic congenital heart disease. Circulation.

[15] Onkelinx S., Cornelissen V., Goetschalckx K., Thomaes T., Verhamme P., Vanhees L. (2012). Reproducibility of different methods to measure the endothelial function. Vasc. Med..

[16] Liu J., Wang J., Jin Y., Roethig H.J., Unverdorben M. (2009). Variability of peripheral arterial tonometry in the measurement of endothelial function in healthy men. Clin. Cardiol..

[17] Reiner B., Oberhoffer R., Häcker A.L., Ewert P., Müller J. (2018). Carotid Intima-Media Thickness in Children and Adolescents with Congenital Heart Disease. Can. J. Cardiol..

[18] Reiner B., Oberhoffer R., Häcker A.L., Ewert P., Müller J. (2020). Is Carotid Intima-Media Thickness Increased in Adults with Congenital Heart Disease?. J. Am. Heart Assoc..

[19] Nichols S., Milner M., Meijer R., Carroll S., Ingle L. (2016). Variability of automated carotid intima-media thickness measurements by novice operators. Clin. Physiol. Funct. Imaging.

[20] Brili S., Tousoulis D., Antoniades C., Aggeli C., Roubelakis A., Papathanasiu S., Stefanadis C. (2005). Evidence of vascular dysfunction in young patients with successfully repaired coarctation of aorta. Atherosclerosis.

[21] Mivelaz Y., Leung M.T., Zadorsky M.T., De Souza A.M., Potts J.E., Sandor G.G. (2016). Noninvasive Assessment of Vascular Function in Postoperative Cardiovascular Disease (Coarctation of the Aorta, Tetralogy of Fallot, and Transposition of the Great Arteries). Am. J. Cardiol..

[22] Goldstein B.H., Golbus J.R., Sandelin A.M., Warnke N., Gooding L., King K.K., Donohue J.E., Gurney J.G., Goldberg C.S., Rocchini A.P. (2011). Usefulness of peripheral vascular function to predict functional health status in patients with Fontan circulation. Am. J. Cardiol..

[23] de Groot P.C., Thijssen D., Binkhorst M., Green D.J., Schokking M., Hopman M.T. (2010). Vascular function in children with repaired tetralogy of Fallot. Am. J. Cardiol..

[24] Laughlin M.H., Newcomer S.C., Bender S.B. (2008). Importance of hemodynamic forces as signals for exercise-induced changes in endothelial cell phenotype. J. Appl. Physiol..

[25] Niwa K., Perloff J.K., Bhuta S.M., Laks H., Drinkwater D.C., Child J.S., Miner P.D. (2001). Structural abnormalities of great arterial walls in congenital heart disease: Light and electron microscopic analyses. Circulation.

[26] Voss C., Duncombe S.L., Dean P.H., de Souza A.M., Harris K.C. (2017). Physical Activity and Sedentary Behavior in Children With Congenital Heart Disease. J. Am. Heart Assoc..

[27] Muller J., Hess J., Hager A. (2012). Daily physical activity in adults with congenital heart disease is positively correlated with exercise capacity but not with quality of life. Clin. Res. Cardiol..

[28] Häcker A.L., Reiner B., Oberhoffer R., Hager A., Ewert P., Müller J. (2018). Increased arterial stiffness in children with congenital heart disease. Eur. J. Prev. Cardiol..

[29] Hock J., Hacker A.L., Reiner B., Oberhoffer R., Hager A., Ewert P., Muller J. (2019). Functional outcome in contemporary children and young adults with tetralogy of Fallot after repair. Arch. Dis. Child..

